# Sleeping Duration, Napping and Snoring in Association with Diabetes Control among Patients with Diabetes in Qatar

**DOI:** 10.3390/ijerph18084017

**Published:** 2021-04-12

**Authors:** Hiba Bawadi, Asma Al Sada, Noof Al Mansoori, Sharifa Al Mannai, Aya Hamdan, Zumin Shi, Abdelhamid Kerkadi

**Affiliations:** Department of Human Nutrition, College of Health Sciences, QU-Health, Qatar University, Doha 2713, Qatar; aa1603830@student.qu.edu.qa (A.A.S.); na1604572@student.qu.edu.qa (N.A.M.); sa1601527@student.qu.edu.qa (S.A.M.); ayahamdan@qu.edu.qa (A.H.); zumin@qu.edu.qa (Z.S.); abdel.hamid@qu.edu.qa (A.K.)

**Keywords:** diabetes, sleep, Qatar biobank

## Abstract

Background: Poor glycemic control is associated with chronic life-threatening complications. Several studies have revealed that sleep status is associated with glycemic control. Aim: to examine the association between sleep duration, quality and glycemic control among adults with diabetes. Methods: Data on 2500 participants aged 18–60 years were collected from the Qatar Biobank (QBB). Sleep duration and quality were assessed by a self-completed health and lifestyle questionnaire, and glycemic control was assessed using HbA1c. Logistic regression was used to assess the association between sleep duration, napping, snoring and poor glycemic control. Results: After adjusting for age and gender, sleep duration was not associated with poor glycemic control. Lack of association persisted after controlling for smoking, physical activity, education, BMI, fruit and vegetable intake, insulin and medication use. However, sleeping for long hours at night (≥8 h) had a trend in increasing the risk of poor glycemic control (OR = 1.28; 95% CI: 0.94–1.74). Napping was positively associated with poor glycemic control. After adjusting for age and gender, patients who reported “sometimes, frequently, or always” napping had more than 30% increased risk of poor control as compared to patients who reported “never/rarely” napping. Snoring was not associated with poor glycemic control among the study sample when adjusted for age and gender (*p* = 0.61). Other factors were found to be associated with a better glycemic control such as female, high educational and high physical activity level. Conclusions: our results suggest that napping may be an independent risk factor for a poor glycemic control in diabetes; further investigations are required.

## 1. Introduction

Diabetes mellitus (DM) is a chronic metabolic disorder characterized by an increase in blood glucose levels resulting from pancreatic defects in insulin production, insulin function, or both. DM has become one of the major public health problems worldwide with more than 350 million estimated to have diabetes and a further eight million people are assessed as prediabetic [1]. The prevalence of diabetes has been increasing rapidly in the Middle East countries for both genders, reaching 14.6% with 81 million individuals in the region being diabetic [2]. The prevalence of diabetes in Qatar among adults has reached 16.5% and the burden is higher among Qataris compared to non-Qataris. The most affected age groups were between 38 and 47 years old (27.9%) followed by 28 to 37 years old (25.5%) [3]. 

Multiple life-threatening complications are associated with diabetes [4]. Uncontrolled diabetes affects patients’ quality of life [4]. However, it has been shown in previously published studies that regular blood glucose monitoring and sustainable glycemic control can prevent and/or delay diabetes complications [5]. Huge body of research documented the impact of multiple lifestyle factors on glycemic control including diet, physical activity and sleeping pattern [6,7,8]. Sleep hygiene is important for the overall health, which implies adequate duration, appropriate timing, good quality, regularity and lack of sleep disturbances [9,10]. Along with the rising epidemic of DM and obesity, the prevalence of sleep disorder has been increasing dramatically worldwide and is considered now as a public health epidemic [11,12,13]. In fact, insufficient sleep time and poor sleeping patterns were associated with attention, behavior and learning issues, with a higher risk of accidents, injuries, hypertension, obesity, diabetes, and depression [10]. Several studies elucidated the interrelation between sleep conditions and some chronic diseases such as diabetes, kidney disease, metabolic syndrome, hypertension, and inflammatory bowel disease [14,15,16,17,18]. Recent meta-analysis indicated that short sleep duration was significantly associated with increased prevalence of MetS (OR 1.11, 95% CI 1.05–1.18) and incident of MetS (RR 1.28, 95% CI 1.07–1.53) in cross-sectional and longitudinal studies, respectively [17]. Another meta-analysis reported a U-shaped association between sleep duration and high risk of hypertension [19]

The finding of diverse studies shows that sleep disturbances, including insomnia, obstructive sleep apnea (OSA), and restless legs syndrome (RLS), are highly prevalent in people with diabetes and ranges from 38 to 97% among people with type 2 diabetes mellitus (T2DM) [13,20]. Moreover, various studies investigated the association between sleep (duration and quality), and glycemic control in diabetic patients [12,15,16,21,22,23].

Sleep duration is suggested to be related to glycemic control in people with diabetes. The recommended duration for healthy sleep for adults is 7 or more hours [24]. The bidirectional relationship between sleep disorders and metabolic disease has been reported by several studies [14,25,26]. Among sleep disorder, Obstructive sleep apnea (OSA) has received a particular attention due to the fact of its relationship with T2DM. Recent studies have elucidated the bidirectional association between OSA and T2DM [25,27]. Results of the population-based study reported that participants with OSA had an estimated hazard ratio (HR, 95% CI) of 2.06 (1.86, 2.28) for diabetes compared to participants without OSA. The same study revealed that the multivariable HR (95% CI) for OSA was 1.53 (1.32, 1.77) in individuals with diabetes [27] (Huang et al., 2018). Hypoxia was suggested to be one possibly underlying mechanism of the relationship between OSA and T2DM [25].

Shorter sleep duration was associated with poor glycemic control and higher glycated hemoglobin (HbA1c) [12,21,28]. Individuals with diabetes who slept less than 5 h/night had higher HbA1c than those who slept 7–8 h/night [28]. Besides, a very short and long sleep durations were positively associated with T2DM [12]. A study found that participants with a sleep duration of ≥9 h had 79% increased odds of having T2DM and an 88% increased odds of pre-diabetes [29]. Furthermore, controversial results were found between napping and diabetes. Some studies showed that daytime nap was associated with a higher risk of developing diabetes [22,23]. In contrast, recent data showed that napping is associated with a better glycemic control in T2DM [12]. In addition, in terms of sleep quality, sleep disturbances have been related to diabetes control. Poor quality of sleep was independently associated with poor glycemic control [30]. However, another study, including participants with type 1 diabetes mellitus (T1DM), showed that the average of glycemic control was negatively linked to sleep quality [21].

To our best knowledge, no study has been conducted in Qatar, where the climate and lifestyle are different, investigating the relationship between sleep patterns and glycemic control. Hence, the present study aims to investigate the association between sleep duration, sleep quality, and glycemic control (i.e., HbA1c) among adult patients with diabetes in Qatar.

## 2. Methods

### 2.1. Study Population

Participants’ data were collected from Qatar Biobank (QBB). QBB is an initiative launched to make vital health research possible by gathering biological samples and information on the health and lifestyle from large numbers of Qatar’s population.

The study population included a case-control of 2500 Qatari adults (men and women) and long-term residents (individuals living in the country for ≥15 years) from 18–60 years of age with a history of type 2 diabetes. Exclusion criteria were pregnant women and patients with terminating illnesses. Participants were categorized into two groups. The control group included participants with good glycemic control (HbA1c < 7%), while the case group included participants with poor glycemic control (HbA1c ≥ 7%). Pregnant women and people with terminating illnesses were excluded. Biochemical, clinical and anthropometric data were obtained from Qatar biobank. It included (HbA1c), fasting plasma glucose tests, blood pressure measurement, and patient’s anthropometrics. In addition to a computer-administered health and lifestyle questionnaire that evolved detailed questions on socio-demographic factors, current and past health, family history of health conditions, current and past smoking habits, occupational information, mobile phone use, physical activity levels, sleeping patterns, reproductive health (women), and cognitive and psychological state. Data of food frequency questionnaire was also provided which included how often participants consume various foods and beverages and any pat modifications to their diet over preceding year. The Qatar Biobank study was approved by the Institutional Review Board from the Hamad Medical Corporation Ethics Committee. The current analysis was approved under the IRB exempted category.

### 2.2. Study Variables

#### 2.2.1. Dependent Variable (Glycemic Control)

The dependent variable of this study was the glycemic control among patients diagnosed with diabetes. The HbA1c was used to assess the glycemic control among the participants in this study. Blood samples were withdrawal from participants and analyzed by biobank in Doha. Based on the American Diabetes Association, the criteria for diabetes was a level of HbA1c ≥ 6.5% (48 mmol/mol) [31]. In the current study, concentrations of HbA1c ≥ 7% were used as an indicator of poor glycemic control.

#### 2.2.2. Independent Variable (Sleep Duration and Quality)

Sleep duration refers to the total amount of sleep obtained either during the nocturnal sleep episode or across a period of 24-h. Sleep duration was determined by the following question “In a typical week during the last year, approximately how many hours of sleep did you get in 24 h? (include naps)”. The answers were categorized into 4 categories: Less than 5 h, between 5 and less than 7 h, between 7 and less than 8 h, 8 h or more. Naps frequency was also assessed and categorized as never/rarely, sometimes, frequently, or always. Snoring was assessed by asking the participants “During the last year, has your spouse or a relative mentioned your snoring? (1) Yes (2) No”.

### 2.3. Covariates

Covariant factors include age, gender, educational level, body mass index (BMI), physical activity level, coffee intake, smoking status, and use of medication. The educational level was divided into three categories: Low level of education (up to secondary school), medium education (technical or professional school), and high education (university and above). BMI was calculated as weight/height squared (kg/m^2^). Participants were categorized into three groups: obese, overweight, and non-obese. Subjects with BMI between 18.5 and 24.9 kg/m^2^ were considered normal, subjects with BMI between 25 and 29.9 kg/m^2^ were considered overweight, and subjects with BMI > 30 kg/m^2^ were considered obese. In terms of lifestyle factors such as physical activity, participants were classified into subgroups (Tertiles) based on their metabolic equivalent (MET) calculation. Qatar biobank provided the participants with self-completed health and lifestyle questionnaires to complete information about their physical activities as well as other factors [32]. After that, Qatar biobank calculated the MET of each participant according to the type and time spent exercising. Only leisure time of physical activity was considered in the calculations. Participant identification of smoking status was provided by Qatar biobank through self-completed health and lifestyle questionnaires that were provided to the participant [32]. In our study, participants were divided into groups as current smokers, history of smoking (ex- smokers) or non-smokers. In terms of dietary intake, coffee consumption was assessed through the self-completed diet questionnaire that was provided by Qatar biobank, participants determined the type of drink (tea/coffee), amount of sugar added, and frequency [32]. Diabetes treatment was assessed by the following question: “Are you being treated for your diabetes?”. Participants can select more than one answer. Answers included no, tablets, insulin, diet, or increased physical activity.

### 2.4. Statistical Analysis

Sample characteristics were presented as mean (SD) or percentage by sleep duration. Chi-square test or ANOVA were used to test the differences between groups for categorical or continuous variables. Logistic regression was used to assess the association between sleep duration, napping, snore and glycemic control. A set of models were used. The first model was adjusted for age and gender; the second model further adjusted for smoking, physical activity, education, BMI, fruit and vegetable intake; the final model further adjusted for insulin use, other diabetes medication, and hypertension medication. We used ordinal number of sleep related measures to test linear trend in multivariable models. All the analyses were conducted using STATA 16 (StataCorp, College Station, TX, USA). Statistical significance was considered when *p* < 0.05.

## 3. Results

### 3.1. Sample Characteristics

Table 1 shows the study sample characteristics by sleep duration. About half of the sample had poor glycemic control and an average age of 51 years. About 41% of the sample were men with about 40% of the study population had either low or high education. The majority of the study sample (74%) were non- smokers. Intake of fruits and vegetables and BMI did not differ according to sleep duration. About 60.6% of the study sample were obese with 25.4% under insulin use and more than half reported taking diabetes medication other than insulin and hypertension medication (34.5%). Around half of the study population snore. Napping was common among the participants with 19.2% and 19.4% reported napping frequent and always. Only gender, educational level and napping were associated with sleep duration (*p* < 0.001). The majority of participants who slept < 5 h or > 8 h had a low educational level (47.5% and 42%, respectively) and those who slept > 8 h always napped (36.6%). The majority of participants that slept between 5 and less than 8 h had higher educational level.

#### 3.1.1. Sleep Characteristic and Poor Glycemic Control

Table 2 represents the logistics regression models for the association between sleep characteristic and poor glycemic control among people with diabetes. After adjusting for age and gender, sleep duration was not associated with poor glycemic control. Lack of association persisted after controlling for smoking, physical activity, education, BMI, fruit and vegetable intake, insulin and medication use. Napping seemed to be positively associated with poor glycemic control. After adjusting for age and gender, patients who reported “always” napping had 37% increased risk of poor control as compared to patients who reported “never/rarely” napping (CI: 1.05–1.78). Results were attenuated after adjusting for smoking, physical activity, education, BMI, fruit and vegetable intake, insulin and medication use. Snoring was not associated with poor glycemic control among the study sample.

#### 3.1.2. Sleep and Other Lifestyle Factors in Relation to with Poor Glycemic Control

**Figure 1:**
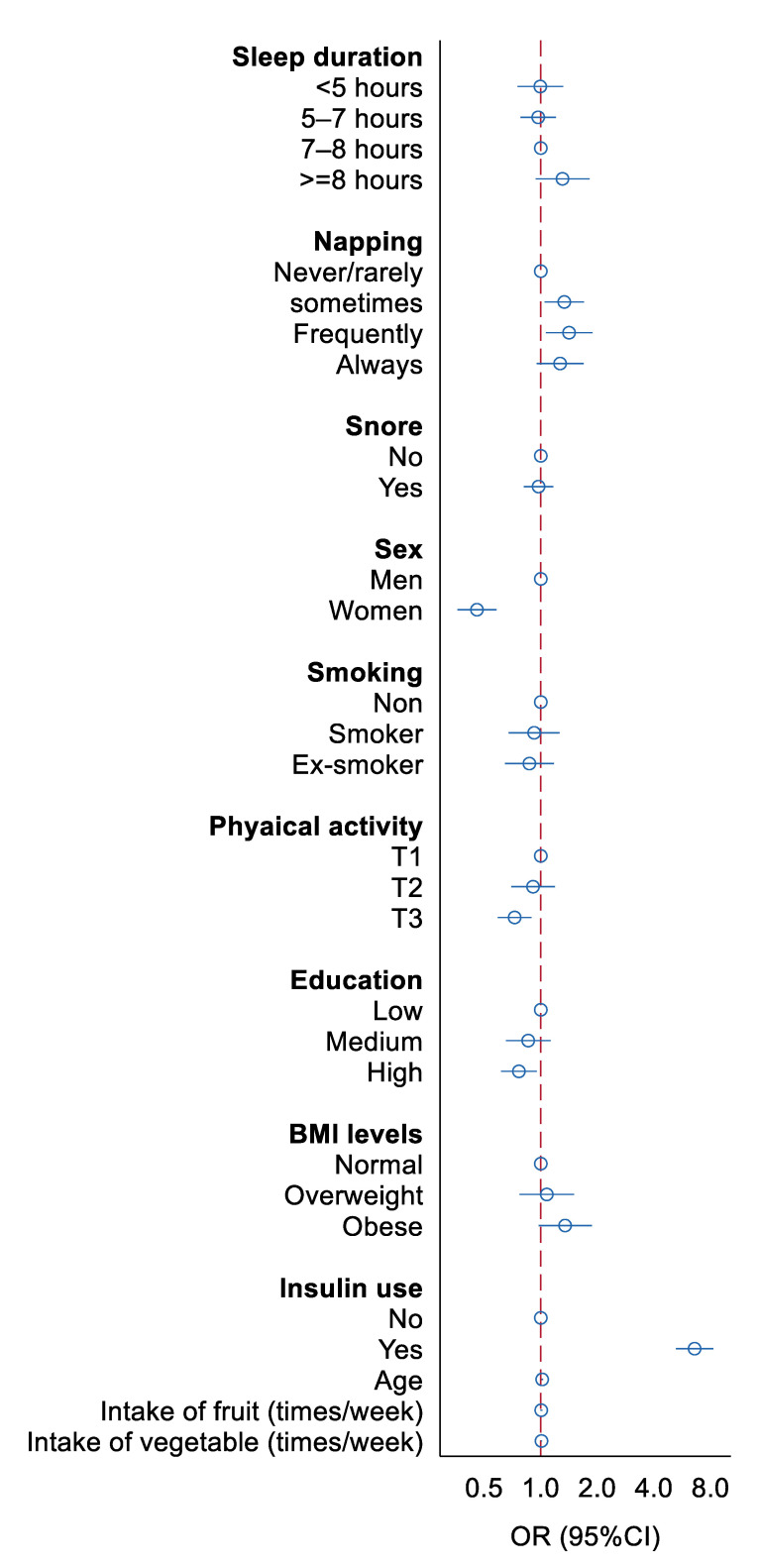
Association between sleep and other lifestyles with poor glycemic control. All the variables were mutually adjusted in the model.

Figure 1 shows the association between sleep and other lifestyles with poor glycemic control. Sleeping for long hours at night (≥8 h) has a trend in increasing the risk of poor glycemic control. Frequent napping was associated to an increased risk of poor glycemic control compared to those who never/rarely napped. Snoring and smoking were not associated with poor glycemic control. Women were less likely to have poor glycemic control than men. Physical activity was inversely associated with glycemic control. Education was inversely but obesity and insulin users were positively associated with poor glycemic control.

## 4. Discussion

The present study demonstrated that diabetic patients napping showed significantly higher HbA1c levels than those who never napped. Additionally, educational level, gender, and napping were associated with sleep duration. These results are consistent with previous findings and indicate that napping has an impact on the glycemic control [22,33,34,35,36].

Several studies had consistently shown that naps could be associated with higher risk of developing diabetes. Napping and long daytime napping were positively associated with diabetes [22,33,34]. The present study found a positive association between napping and poor glycemic control. Similar results were reported by other studies, showing a linear relationship [22,34]. In a prospective Cohort study, Li et al. reported that daytime napping >30 min was associated with an elevated HbA1c level. In addition, no sleep deprivation combined with napping >30 min carries a risk of abnormal glucose metabolism. Whereas, sleep deprivation combined with brief daytime napping <30 min was not associated with an elevated HbA1c level [37]. In contrast, a study showed that naps could relieve the side effects of short nighttime sleep on glycemic control in T2DM [38]. Some studies did not find an association between napping and glycemic control among type 2 diabetes cases [12,38]. Findings revealed several potential inter-related sleep and circadian mechanisms in which napping could affect the glycemic control [35,36]. Both sleep homeostatic and circadian processes have profound influences on multiple physiological functions including the release of pancreatic insulin, insulin sensitivity and sympathetic-parasympathetic balance [39,40]. Increasing sympathetic activity upon awakening from naps, mainly prolonged naps, could results in disruption of the sympathovagal balance, activation of the renin-angiotensin system, which can minimize pancreatic beta-cell insulin secretion, insulin resistance and associated hyperglycemia [36,41,42]. Another proposed mechanism stated that people who nap might be partly less active. This results in reciprocal changes in circulating levels of leptin and ghrelin, which might increase appetite and caloric intake, reduce energy expenditure, and lead to impaired glycemic control [43]. In our study, the correlation demonstrated between napping and poor glycemic control was still significant after adjusting for the confounding factors. Worthwhile mentioning, napping may be a surrogate marker for obstructive sleep apnea which is an established risk factor for poor glycemic control in patients with diabetes. No data about sleep apnea was available; hence, the association between napping, sleep apnea and glycemic control was not possible.

In addition, our results showed that the majority of participants who slept for <5 h or from 5–8 h napped less compared to participants who slept longer at night (>8 h). The majority of participants who slept >8 h always napped (36.6%). Napping is considered desirable to compensate for the negative impact of insufficient nighttime sleep on health, especially in short sleepers (<5 h) [44,45,46]. Our findings need further investigation about the quality of sleep of those that slept longer at night especially that the proportion of participants snoring was not different than the other groups.

The accumulated evidence suggests that sleep disturbances as well as altered sleep duration is associated with higher HbA1c levels [47,48,49]. In the present study, sleep duration did not seem to significantly affect HbA1c levels. However, participants sleeping more than 8 h tend to have a higher HbA1c. Previous studies reported that short and long sleep durations were related to high HbA1c levels in diabetic patients [47,48]. Lee SWH, et al. ([9]) found similar results in T2DM participants, with a U-shaped dose-response relationship. The mechanism is still not well understood but some studies suggest that the correlation is mediated by the impact of sleep duration on appetite-regulating hormones [50]. Short sleep duration is believed to be related to higher levels of ghrelin hormone and decreased leptin hormone level, which could potentially lead to increased appetite, food intake, obesity, and impaired glucose tolerance [47,48]. However, the mechanism of long sleep duration and high glycemia is still unclear and needs further investigation [48].

Furthermore, habitual snoring is highly prevalent, especially among diabetic patients [51,52,53]. Obstructive sleep apnea (OSA) including snoring was independently associated with diabetes and higher HbA1c values [34,54]. In our study, we did not find any relation between snoring and glycemic control. Other studies have found similar results with an association on the borderline of statistical significance [18]. The mean HbA1c among Type 1 diabetes miletus (T1DM) patients was similar between people who snore and those who did not [55]. Nevertheless, studies revealed that the severity of obstructive sleep apnea syndrome (OSAS) was associated with glycemic control. A higher snoring intensity and frequency were positively associated with glycemic control [56]. The association between snoring and T2DM is biologically plausible [57]. Possible mechanisms were discussed. Since snoring inhibits good quality sleep through oxygen desaturation and upper airway obstruction, it could lead to insulin resistance and disruption of glucose metabolism [57,58]. Increased sleep fragmentation and frequent arousals trigger pro-inflammatory cascade by elevating interleukin-6, C-reactive protein, and fibrinogen levels and lowering albumin levels, thereby systematically damaging glucose stability and beta-cell function [58]. Another mechanism proposed in several studies, that snoring activates the sympathetic nervous system, resulting in catecholamine elevation and hypothalamic-pituitary-adrenal axis activation. This combination of elevated cortisol, formation of reactive oxygen species, and increased oxidative stress alter sleep thereby leads to impaired glucose metabolism [56,59,60,61,62]. Moreover, the intermittent hypoxia and marked sleep fragmentation often cause excessive daytime sleepiness [56]. Thus, in the current study, snoring intensity and frequency may have confounded the association between nighttime sleep duration, midday naps, and glycemic control.

A number of factors such as educational level, body mass index (BMI), gender, physical activity, smoking, and insulin use may confound the association between sleep patterns and glycemic control. In terms of education, several studies found a significant impact of the educational level on the glycemic control [63]. Participants with higher educational level had a better awareness of the complications and a high rate of adherence to diet [63]. In the present study, we did not find a significant correlation between the educational level and glycemic control. However, participants with a high educational level tend to have better glycemic control. These results are consistent with other studies [64,65]. Educational level was significantly associated with better attitudes and practices that helps in controlling diabetes [64,65,66,67,68]. A recent study revealed that people with educational qualifications had good knowledge regarding diabetes compared to those who could not read and write [66]. The HbA1c levels were highest among low-educated adults, and death risks associated with uncontrolled HbA1c were significantly greater [67]. Good glycemic control was observed among type 2 diabetic Pakistani patients who scored higher for diabetes knowledge [68]. Conversely, another study found that disease knowledge was not significantly correlated with HbA1c levels [69].

Body mass index (BMI) is one of the important factors for measuring obesity. In our study, obesity had a trend to increase the risk of poor glycemic control. The majority of the study population were obese. A study showed a positive association between obesity and having a suboptimal glycemic control (HbA1c level ≥ 7%) in both T1DM and T2DM [70]. However, a case-control study analyzing the association between BMI and glycemic control showed that having higher BMI (>25 kg/m^2^) was not associated with greater odds of having a higher HbA1c. They compared patients who had suboptimal glycemic control defined by an HbA1c value >7% with patients who had optimal glycemic control defined by an HbA1c value <7% [71]. Also, our analysis showed that physically active participants tend to have better glycemic control. The latter can be explained by the process of glucose uptake by skeletal muscles during exercise. Glucose is transported from the capillary bed via facilitated diffusion through the GLUT4 transporter as an energy substrate for the working muscle during exercise [72].

The present study revealed that women tend to have less risk of poor glycemic control compared to men. In contrast, a cross-sectional study including 9418 patients with T2D found that women with T2DM had worse glycemic control than men due to the differences in glucose homeostasis, treatment response, and psychological factors [73]. In 2015, a study including 602 adult patients with T2DM shows that gender was not significantly associated with poor glycemic control in either unadjusted or adjusted analyses [74]. Furthermore, there was no association between smoking and poor glycemic control. However, in other studies smoking was positively associated with poor glycemic control due to the presence of neuronal nicotinic acetylcholine receptors (nAChRs) in the β cells of pancreatic islets, which affects the insulin secretion [75,76]. Moreover, in the present study, insulin use was associated to a higher risk of poor glycemic control. Similarly, a cross-sectional study including 1253 patients with T2DM revealed an increased risk for poor glycemic control in patients using insulin. Possible reasons were storage conditions and improper usage of insulin. They found that only 37.6% of the participants used self-monitoring blood glucose devices, which indicates a lack of proper monitoring [77]. However, studies have shown that intensive insulin therapy in type 2 diabetic patients results in excellent glycemic control as it improves insulin sensitivity and regulates blood glucose levels [78].

This study offers several strengths. The sample size is large and multiple variables were taken into consideration such as age, gender, education, smoking, and BMI. Nevertheless, our study has some limitations. First, the present study had qualitative data about coffee consumption and naps but not quantitative. We did not ask for the details of napping such as whether the nap was voluntary or involuntary, the nap environment (e.g., light and noise level), sleepiness after awakening, and sleep satisfaction. Second, the characteristics of the study population including male/female ratio among the study sample may be a source of discrepancy in results interpretation as women were predominant. In addition, more than half of the population were obese and this could also affect the results interpretation. Another limitation of the current study may be the assessment of the sleep quality without dedicated questionnaire (PSQI). Future study may need to verify the results of this study using objective sleep assessment tool such as actigraphy. Finally, there was insufficient information regarding napping time and snoring intensity and frequency, which may affect the association between napping and glycemic control. Despite the limitations of the study, we found a significant association between napping and the risk of poor glycemic control.

## 5. Conclusions

In conclusion, our results suggest that sleep health is an important modifiable risk factor for improving glycemic control in diabetes. Napping may be an independent risk for poorer glycemic control. Further investigation is definitively necessary to determine whether napping is beneficial or not for improving glycemic control, taking into account the frequency and intensity of snoring. Further research is needed to establish the causal link between sleep and impaired glucose metabolism. These findings may open up new strategies for targeted intervention to improve the duration and quality of sleep.

## Figures and Tables

**Table 1:** Sample characteristics by sleep duration.

	Total	<5 h	5–7 h	7–8 h	≥8 h	*p*-Value
	**N = 2448**	**N = 406**	**N = 1182**	**N = 617**	**N = 243**	
Poor glycemic control	1250 (51.1%)	204 (50.2%)	598 (50.6%)	311 (50.4%)	137 (56.4%)	0.38
Age	51.6 (11.9)	52.1 (11.5)	51.6 (11.7)	51.8 (11.8)	50.5 (13.4)	0.43
Gender						<0.001
Men	1000 (40.8%)	153 (37.7%)	531 (44.9%)	219 (35.5%)	97 (39.9%)	
Women	1448 (59.2%)	253 (62.3%)	651 (55.1%)	398 (64.5%)	146 (60.1%)	
Education						<0.001
Low	982 (40.1%)	193 (47.5%)	442 (37.4%)	245 (39.7%)	102 (42.0%)	
Medium	461 (18.8%)	91 (22.4%)	218 (18.5%)	108 (17.5%)	44 (18.1%)	
High	1004 (41.0%)	122 (30.0%)	521 (44.1%)	264 (42.8%)	97 (39.9%)	
Smoking						0.20
Non	1820 (74.3%)	301 (74.1%)	857 (72.5%)	484 (78.4%)	178 (73.3%)	
Smoker	296 (12.1%)	53 (13.1%)	149 (12.6%)	62 (10.0%)	32 (13.2%)	
Ex-smoker	332 (13.6%)	52 (12.8%)	176 (14.9%)	71 (11.5%)	33 (13.6%)	
Leisure time physical activity (MET hours/week)	0.0 (0.0–12.0)	0.0 (0.0–8.0)	1.5 (0.0–15.0)	0.0 (0.0–10.5)	0.0 (0.0–12.0)	<0.001
Intake of fruit (times/week)	7.5 (4.0–14.0)	7.5 (4.0–14.0)	7.5 (4.0–14.0)	7.5 (4.0–14.0)	7.5 (4.0–14.5)	0.83
Intake of vegetable (times/week)	18.0 (9.5–27.0)	17.0 (8.5–28.0)	18.0 (9.5–27.0)	19.0 (10.5–27.5)	18.0 (10.0–27.5)	0.42
Leisure time physical activity (MET hours/week)	12.6 (36.4)	12.3 (56.9)	13.7 (33.9)	10.9 (22.4)	12.3 (32.2)	0.50
Intake of fruit (times/week)	9.7 (7.9)	10.3 (9.2)	9.5 (7.6)	9.7 (7.4)	9.6 (7.9)	0.33
Intake of vegetable (times/week)	20.6 (15.6)	21.0 (18.0)	20.4 (14.7)	21.3 (16.0)	19.9 (14.5)	0.55
BMI (kg/m^2^)	32.1 (6.0)	32.5 (6.7)	31.9 (5.7)	32.1 (5.8)	32.3 (6.3)	0.31
BMI categories						0.67
Normal	212 (8.7%)	40 (9.9%)	103 (8.7%)	49 (8.0%)	20 (8.2%)	
Overweight	751 (30.7%)	113 (27.9%)	369 (31.3%)	186 (30.2%)	83 (34.2%)	
Obese	1481 (60.6%)	252 (62.2%)	708 (60.0%)	381 (61.9%)	140 (57.6%)	
Insulin use	621 (25.4%)	96 (23.6%)	305 (25.8%)	155 (25.1%)	65 (26.7%)	0.80
Diabetes medication other than insulin	1705 (69.6%)	266 (65.5%)	831 (70.3%)	443 (71.8%)	165 (67.9%)	0.16
Hypertension medication use	844 (34.5%)	146 (36.0%)	407 (34.4%)	202 (32.7%)	89 (36.6%)	0.63
Snore	1253 (51.3%)	214 (53.1%)	605 (51.3%)	308 (50.1%)	126 (51.9%)	0.82
Nap						<0.001
Never/rarely	474 (19.4%)	113 (27.8%)	221 (18.7%)	106 (17.2%)	34 (14.0%)	
Sometimes	1030 (42.1%)	187 (46.1%)	523 (44.2%)	239 (38.7%)	81 (33.3%)	
Frequently	470 (19.2%)	48 (11.8%)	236 (20.0%)	147 (23.8%)	39 (16.0%)	
Always	474 (19.4%)	58 (14.3%)	202 (17.1%)	125 (20.3%)	89 (36.6%)	

Data are presented as mean (SD) or median (IQR) for continuous measures, and n (%) for categorical measures.

**Table 2:** Association (OR 95% CI) between sleep characteristic and poor glycemic control among people with diabetes.

**Sleep Duration**	**<5 h**	**5–7 h**	**7–8 h**	**>8 h**	***p* for Linear Trend**
Model 1	0.98 (0.76–1.26)	0.97 (0.80–1.18)	1.00	1.30 (0.96–1.75)	0.13
Model 2	0.93 (0.72–1.20)	0.98 (0.80–1.20)	1.00	1.28 (0.94–1.74)	0.09
Model 3	1.06 (0.80–1.40)	0.98 (0.79–1.22)	1.00	1.34 (0.96–1.88)	0.28
**Napping**	**Never/Rarely**	**Sometimes**	**Frequently**	**Always**	***p* for Linear Trend**
Model 1	1.00	1.37 (1.10–1.71)	1.32 (1.02–1.72)	1.37 (1.05–1.78)	0.058
Model 2	1.00	1.38 (1.10–1.73)	1.36 (1.04–1.77)	1.37 (1.05–1.78)	0.055
Model 3	1.00	1.29 (1.01–1.66)	1.38 (1.04–1.85)	1.26 (0.94–1.69)	0.14
**Snoring**	**No**	**Yes**		***p* Values**	
Model 1	1.00	0.96 (0.82–1.13)		0.61	
Model 2	1.00	0.93 (0.79–1.10)		0.40	
Model 3	1.00	0.97 (0.81–1.16)		0.71	

Model 1: adjusted for age and gender. Model 2: further adjusted for smoking, physical activity, education, BMI, fruit and vegetable intake. Model 3: further adjusted for insulin use, other diabetes medication, hypertension medication.

## Data Availability

Data is owned by Qatar BioBank and is not an open source data.
